# Outcome of mitral valve plasty or replacement: atrial fibrillation an effect modifier

**DOI:** 10.1186/1749-8090-8-142

**Published:** 2013-06-01

**Authors:** Øystein A Vengen, Michael Abdelnoor, Arne S Westheim, Gunnar Smith, Nils Bj Fjeld

**Affiliations:** 1 Dept of Cardiothoracic Surgery, Oslo University Hospital, Ullevaal, Norway; 2 Centre of Clinical Research: Unit of Epidemiology and Biostatistics, Oslo University Hospital, Ullevaal, Norway; 3 Centre for Clinical Heart Research, Oslo University Hospital, Ullevaal, Norway; 4 Dept of Cardiology, Oslo University Hospital, Ullevaal, Norway

**Keywords:** Mitral plasty, Mitral replacement, Mortality, Reoperation, Atrial fibrillation

## Abstract

**Background:**

Advances in the understanding of mitral valve pathology have laid to mitral valve plasty (MPL) as the procedure of choice of all the mitral intervention as compared to mitral valve replacement (MVR).

This study is aimed to compare the outcome mortality and reoperation and to estimate failure of repair between the two procedures during the follow up time.

**Material and methods:**

A cohort of 355 patients with mitral valve disease operated between January 1993 to January 2007 with closing date first of mars 2011. There were 214 MPL and 141 MVR at the Hospital discharge. This retrospective cohort had the design of exposed (MPL) versus non-exposed (MVR) with outcome total mortality and reoperation during follow up. Also echocardiography follow-up was undertaken to estimate the true long-term failure rate of repair.

**Results:**

The mean follow up was 5.3 years SE (3.82) maximum follow up was 14.1 years. Considering the patient time model the association between repair/replacement and total mortality RR = 0.43 95% (0.28-074) p = 0.002 controlling for the confounding effect of 3-vessels disease. Those results were confirmed by propensity score analysis.

As far as outcome re-operation, presence of atrial fibrillation AF was an effect modifier indicating lower reoperation rate for MPL compared to MVR for patients without AF, RR = 0.32 95% CL (0.13-0.81) p = 0.017 while no difference in reoperation rates between MPL/MVR for patients with AF RR = 1.82 95% CL (0.52-6.4) p = 0.344.

Echocardiography follows up showed incidence of moderate and severe recurrent mitral regurgitation was 1.34 per 100 patients years and 0.27 per 100 patients years during the follow-up time.

**Conclusion:**

In a cohort of patient with mitral valve disease undergoing MPL/MVR was examined. MPL was associated with better survival, and lower reoperation rate for patients without AF but same rate for patients with AF. We advocate more attention in controlling risk factors of AF in the clinical management of mitral disease. Long-term failure rate of MPL was low during follow up time. A replication of our results by a randomized clinical trial is mandatory.

## Background

Advances in the understanding of mitral valve pathophysiology and technology has brought mitral valve plasty (MPL) as the procedure of choice.

MPL has been considered more often for patient with sever impairment of left ventricular systolic function where ischemia was the cause of mitral regurgitation. It is not clear that MPL is superior to MVR in all clinical circumstances. So far, no randomised clinical trial comparing mortality and morbidity after MPL versus MVR has been undertaken. Hence, our information is based on the majority of observational studies showing some evidence for better survival from MPL as compared to MVR [1,2] The risk of thromboembolism was lower in MPL without increasing frequency of re operation for MPL as compared to MVR. Better survival of MPL as compared to MVR has been observed for patients with specific aetiologies (rheumatic, mixed or degenerative). In patients with chronic ischemic regurgitation, there is no consensus in the literature about which method is the best one. Recently Murphy et al. [3] suggested performing a randomised trial to answer this important issue.

Further, sufficient data are not available on failure of MPL defined by recurrence of moderate or severe mitral regurgitation or reoperation for mitral regurgitation. Postoperative incidence of recurrent mitral regurgitation is principally an end-point to evaluate for the long-term outcomes of MPL. In the absence of echocardiographic follow up, there is no way of quantifying the long-term failure rate of repair.

This study was aimed to compare the outcome total mortality and re operation between the two methods during a long-term follow-up (mean follow up time of 5.3 years). Further, we wanted to highlight the durability of MPL by a serial of echocardiography done at discharge, after medium and long term follow up to estimate the rate in100 patients.year of new recurrent mitral regurgitation.

## Methods

A cohort of 355 consecutive patients with mitral valve disease were operated between January 1993 to December 2007 at Ullevaal University Hospital with a longitudinal follow up until the closing date of first of March 2007.

There were 214 MPL and 141 MVR at baseline as primary operation. Twenty procedures were converted intraoperatively from MPL to MVR and were reported as MVR at discharge from hospital. Hence, 214 patients were operated with MPL and 141 with MVR It is important to emphasize that the whole material of 355 mitral operations represent **all** mitral surgical procedures undertaken at this institution in this period. The study had been ethically approved by the Head of the Office of Privacy and Information Security at Oslo University Hospital which is the juridical responsible organism.

### Echocardiography and clinical data

Echocardiography by an experienced cardiologist was performed pre discharge and after 3,12, 36,60,120 months. Measurement of mitral regurgitation was performed according to the American Society of Echocardiography [4]. Follow up data were collected from the outpatient department at the local hospitals and their general practitioners. If no reply was received, the patients were contacted by telephone. Mortality data were retrieved from Statistics Norway (http://www.ssb.no/english/) Autopsy was mostly but not always performed on patients who died in hospital. No patient was lost to follow up.

The follow up time in days ranged from 1 to 5160 days with a mean observation time of 5.3 years SD (3.82), and the total follow up time represented 1859 patients years.

### Surgical considerations

This surgical material consists of all mitral surgical procedures undertaken at this institution in this period (1993–2007). The same 2 surgeons undertook all operations. All operations were undertaken with extracorporeal circulation in moderate hypothermia (32 ^c^ c). Myocardial protection was achieved by antegrad blood cardioplegia. The MVRs aimed to preserve chordal preservations to the largest extent possible. Patients older than 70 years received biological prosthesis (Medtronic Mosaic) and (<70 Years) received St Jude bileaflet mechanical prosthesis.

In the beginning of this observational period, MVR were more frequent procedure than MPL. However, after 1998, about 2/3 of mitral surgical procedures where MPLs.

Different repair procedures were performed in the group of mitral repair MPL. Mitral ring placed (annuloplasty) was done usually combined with other procedures in 187 patients.

145 patients got a posterior leaflet resection, 26 patients got anterior leaflet resection, 16 patients got chordal shortening, 7 patients had artificial chordea, 7 patients got chordal transfer and finally 16 patients got other mitral plasty.

### Before hand power estimate

Our before hand power analysis was done considering our past experience in our centre and information published in the literature. Our centre results [5] as far as 5 years survival of the MVR procedure totally was 78% SE (3.0%) (Cum hazard 22%) and MVR with presence of mitral regurgitation 75.5% SE (4.7%) (Cum hazard 24.5%). On the other hand Cohn LW et al. [6] indicated at 2.5 years of follow up 6% cumulative incidence of mortality for MPL and 15% cumulative incidence for MVR. This gave an RR = 0.40 pinpointing a 60% reduction of mortality for repair as compared with replacement. Another investigator Moss RR et al. [7] considering propensity score matched cohort, indicated at 5 years of follow up a cumulative incidence of mortality of 8% for MPL and 17% for MVR showing an RR = 0.47; i.e. a 53% reduction of mortality in favour of repair.

Considering the mean of the two estimated RR = (0.41 +0.47)/2 = 0.44 (56% reduction of mortality in favour of repair) and a cumulative incidence of mortality at 5 years of 24.5% for MVR [5] an a type I error of 5% and power of 80% and a ratio of MPL/MVR = 2 we will need around 212 MPL patients and 106 MVR patients a total of 318 patients considering 10% dropout.

Gillinov Am et al. [8] pinpointed no difference in the freedom of re operation survival between repair and replacement at 5 years (94% versus 95%) p = 0.60. No power estimated was done as far this end point as there is no difference in re operation rate between repair and replacement [1].

#### Epidemiological design and statistical methods

An observational cohort study design of exposed MPL versus unexposed MVR was considered with outcome total mortality and incidence of re operation as the major endpoints of the study.

To highlight the major confounders and effect modifier of the association MPL versus MVR on the effect parameters mortality and re operation we used the Mantel Hanszel methods. Survival curves using the Kaplan Meier methods were used with end point total mortality and incidence of re operation. The log rank test was used to compare the survival. The Cox’s multivariate model to control for confounders and interactions [9,10].

Finally, we also considered the propensity score (PS). We know that non-randomised comparison gives rise to selection bias. PS is the conditional probability to have a MPL given the individual covariates [11]. Adjusting observed MPL with the probability of MVR (propensity) creates a quasi-randomised experiment. This can be used to adjust for selection bias via design matching or during analysis of treatment effect by stratification on quintiles of the PS in the final Cox’s regression model [12]. The propensity score was based on 6 variables differently distributed between the two treatments as age, gender, ischemic aetiology, atrial fibrillation, ejection fraction, and degenerative aetiology. Our model is in agreement with the limitations by Peduzzi et al. [13] pinpointing the need to have not less than 10 events per covariates. A review of application of propensity score methods [14] has showed that there is little evidence that these methods yield substantially different estimates compared with conventional multivariable method.

As we are in a prognostic strategy in the propensity score estimation analysis, the predictive accuracy of the model was evaluated by calibration and discrimination. Calibration was evaluated by the Hosmer and Lemeshow (H-L) goodness of fit test. Discrimination was evaluated by the analysis of the area under the ROC curve. If the area under the curve is greater than 0.7, it can be concluded that the model has an acceptable discriminatory capability [15]. We assessed the balance in baseline variables between MPL and MVR patients within quintiles of the Propensity Score using the standardized difference method (16,17).

The reporting and analysis of our study followed the suggestions for improvement of [16-18] when combining classical methods and Propensity score to remove 90% of measured selection bias due to baseline difference using quintiles stratification [11]. Unlike randomization, PS techniques cannot control for unknown or unmeasured potential confounding factors.

## Results

### Generating the propensity score

We generated a propensity score by using the logistic model and including 6 variables as ischemic aetiology, degenerative aetiology, EF preoperatively, age at operation, gender, presence of AF preoperatively. The Hosmer-Lemeshow (H-L) test for the model was not significant indicating a useful goodness of fit for the model. The predictors of MPL by constructed logistic model demonstrate a good discriminatory power. Area under the ROC curve (AUC) was 0.819 95% CL (0.775-0.857).

The assessment of distribution of the baseline variables between the two procedures MPL and MVR within quintiles of the PS was satisfactory using the standardized difference method.

### End point mortality

The follow up time inn days ranged from 1 to 5160 days with a mean observation time of 5.3 years SD (3.82) total follow up time represented 1859 patients years.

Patients with MPL as compared to MVR were older, higher frequency of male, less frequency of AF, higher frequency of MR, less frequent endocarditic aetiology, lower frequency of significant CHD, higher ejection fraction and higher frequency of degenerative aetiology (Table 1).

**Table 1:** Clinical profile of patient with mitral plasty and mitral valve replacement

**Variables**	**MPL(n = 214)**	**MVR(n = 141)**
Age yrs	64(54–72)	62(51–69)*
EF in %	60(50–73)	50(50–63)*
Female	59	59
Presence of Dyspnea	201	133
Presence of angina	43	28
Presence of MR	203	127
Mild Grad-I	3	1
Moderate Grad-II	15	27
Severe Grad-III	181	99
Presence of MR + MS	11	14
Presence of AF	54	55
Presence of AR	25	40
Presence of AS	14	26
Presence of TR	30	31
Presence of PR	2	2
3 vessels disease	32	19
Significant CHD	62	43
**Etiology/Pathology**		
Barlow	4	2
Myxoma	1	0
Ischemic MR	29	21
Endocarditis	21	26
Degenerative	120	13
Unknown etiology	46	34
Papillary muscle rupture	10	7
Valve thrombosis	0	1
**Previous valve surgery**		
Previous MVR	2	10
Previous AVR	3	5
Previous plasty	1	3

*MS* Mitral Stenosis, *AF* Atrial Fibrillation, *AS* Aortic Stenosis, *TR*, Tricuspid regurgitation.

*PR* Pulmonary valve regurgitation, *AR* Aortic regurgitation, (*) Median and interquartile interval.

Significant CHD: Major stenosis over 75% in one of the major coronary artery.

**Figure 1:**
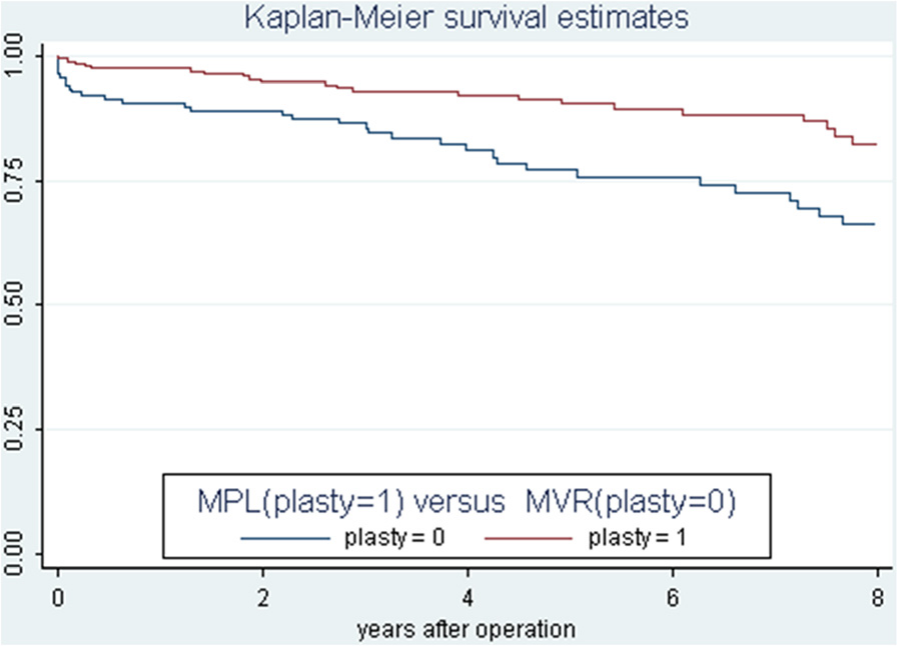
Comparative survival of mitral plasty versus mitral valve replacement.

The 5 years survival for MPL group was 90.6% SE (2.32) and for MVR 75.8% SE (4.28) with log rank test p = 0.001 (Figure 1). Considering the patients years model the association between procedures MPL/MVR and total mortality, was estimated by a Rate Ratio (RR) = 0.48 95% (0.28-0.80) p = 0.001 indicating a relative reduction of mortality of MPL versus MVR of 52%. A stratification analysis using the Mantel-Hanszel methods showed that the strongest confounder was presence of three vessels disease with a confounding effect of 6.2% and no effect modifier. No significant difference in stroke and cerebro vascular bleeding was found between the MPL group and the MVR group but a tendency towards lower risk for MPL as compared to MVR. In this situation we cannot be conclusive as we are in a situation of power deficiency Table 2.

**Table 2:** Crude effect of mitral plasty (MPL) versus mitral valve replacement (MVR) on total mortality and re operation and incidence of stroke and cerebral bleeding at a mean follow up of 5.2 years

**Total mortality and re operation**				
Variables	MPL	MVR	Rate ratio with 95% CL	P-value
Mortality	29	39	0.48(0.28-0.80)	0.001
Patients Years	1125	734		
Re operation	14	17	0.61(0.28-1.31)	0.1775
Patients Years	1149	854		
**Morbidity stroke and cerebral bleeding**				
Stroke	8	10	0.52 (0.18-1.35)	0.1315
Cerebral bleeding	3	3	0.65 (0.08-4.90)	0.6171
Patients years	1125	734		

### Adjusted effect of procedure on total mortality

Controlling for the confounding effect of presence of three vessels diseases we got an adjusted effect of RR = 0.43 95% CL (0.28-0.74) p = 0.002 taking in consideration validity and precision, this indicates a relative reduction of mortality of 57% for MPL as compared to MVR as far as total mortality Table 3.

**Table 3:** Adjusted effect of plasty procedure versus replacement controlling for confounders and level of interaction (effect modification)

**Using the Cox’s regression model**			
**Total mortality**			
Variables	Level	RR with 95% CL	P-value
MPL/MVR	Yes/No	0.43(0.28-0.74)	0.002
3 vessels CHD	Yes/No	2.74(1.59-4.59)	0.0001
**Reopration**			
MPL/MVR	Yes/No	0.32(0.13-0.81)	0.017
AF	Yes/No	0.47(0.15-1.47)	0.198
AF*(MPL/MVR)	Interaction	5.6(1.21-26.6)	0.028
Presence significant CHD	Yes/no	0.47(0.19-1.14)	0.091
**Using the Cox’s model stratified by propensity score quintiles**			
**Total mortality**			
Variables	Level	RR with 95% CL	P-value
MPL/MVR	Yes/no	0.54(0.32-0.90)	0.02
3 V CHD	Yes/no	2.58(1.50-4.43)	0.001
**Reoperation**			
MPL/MVR	Yes/no	0.75(0.32-1.72)	0.502
Sig. CHD	Yes/no	0.46(017–1.21)	0.1190

Interaction between MPL/MVR and AF:

Absence of AF: RR = 0.32 95% (0.13-0.81) p = 0.017.

Presence of AF: RR = 1.82 95% (0.52-6.4) p = 0.344.

As presence of AF is part of the propensity score. Interaction between MREP and AF could not be performed.

Sig. CHD: Presence of significant coronary artery stenosis > 50% in one of the major coronary artery.

Another approach was done by running the stratified Cox’s model based on quintiles of PS (propensity score) and controlling for the variable presence of 3 vessels disease. This analysis gave an RR = 0.54 with 95% CL (0.32-0.90) p = 0.02 indicating a reduction of mortality of 46% MPL as compared to MVR at a mean follow up time of 5.3 years.

### Endpoint re operation

**Figure 2:**
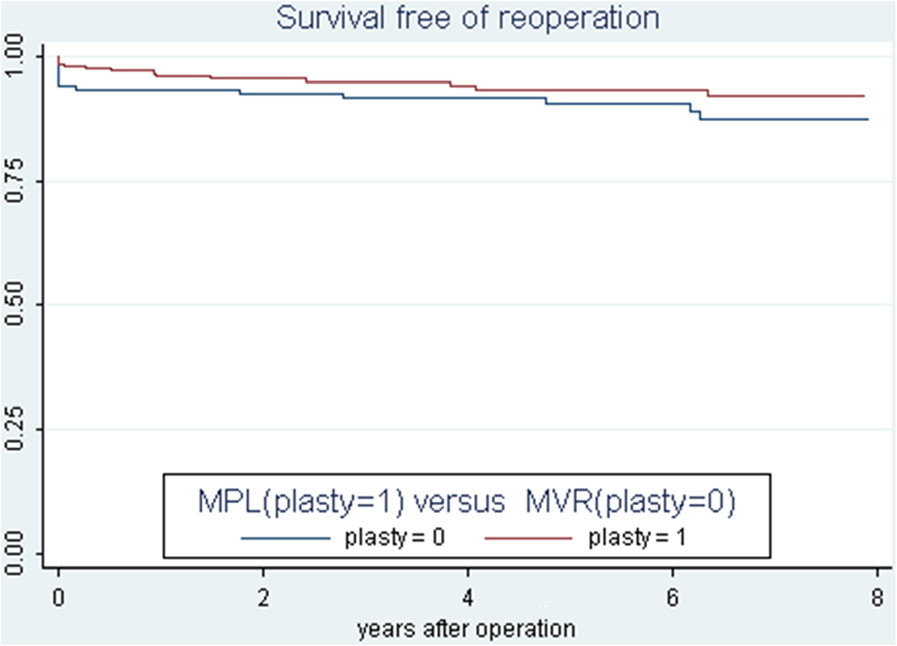
Comparative survival free of re operation between mitral plasty and mitral replacement.

At 5 years of follow up patient with MPL had a cumulative survival free from reoperation of 93.4% SE (1.8%) compared to a survival free from reoperation of 90.6% SE (2.6%) for MVR. p = 0.1518 (Figure 2). A crude effect estimate using the patient time model showed that the association between procedure MPL/MVR and re operation was quantified by RR = 0.58 (0.26-1.26) p = 0.1353 which indicates no difference between the two the procedure regarding the incidence of re operations Table 2.

A stratification analysis using the Mantel-Hanszel methods on the person time model showed that presence of significant CHD was the only variable with confounding effect around 3.3% and the presence of atrial fibrillation (AF) was an effect modifier.

### Adjusted effect of procedure on incidence of re operation

The adjusted effect of MPL/MVR procedure on re operation was done using the Cox’s model and controlling for the confounding effect of presence of significant CHD and the interaction between procedure and presence of AF. Our results are shown in Table 3.

The interaction term continued to be statistically significant (p = 0.028), because of interaction our results can be summarized as follow. For non-AF patients performing MPL will give us an RR = 0.32 95% CL (0.13-0.81) p = 0.017, indicating 68% less re-operation incidence for MPL as compared with MVR. For patients with AF, there was no difference in the incidence of re operation between repair and replacement. In our study the prevalence of AF was around 31% in accordance with the literature [1] and our AF patients were older 68 years (62–73) for AF (+) versus 60 years (50–68) for AF (−) p = 0001, they had a larger left atrium diameter 53 mm (47–58) for AF (+) versus 47 mm (40–53) for AF (−) p = 0.00001 and a lower ejection fraction 56% (50–66) for AF (+) versus 60% (50–76) for AF (−). P = 0.03 (median values and interquartile interval are presented).

Another approach was done by running a stratified Cox’s model based on quintiles of PS and controlling for the confounding effect of the variable presence of significance CHD. This analysis gave an RR = 0.75 with 95% CL (0.32-1.72) p = 0.502 indicating no difference in re operation incidence for MPL as compared to MVR, at a mean follow up time of 5.3 years postoperatively. Investigating the interaction between presence of AF and the procedure considered was impossible to perform as presence of AF was one of the component variables of the propensity score.

### Residual mitral regurgitation rate estimated by echocardiography during follow up

The efficacy and durability of repair was estimated by echocardiography on discharge and by a serial of echocardiographic examinations performed at 3,12,36,60 and 120 months of follow up. This permitted us to estimate the rate of new recurrent mitral regurgitation in events per 100 patients years. The results are shown in Table 4, the failure to repair is defined by recurrence of moderate to severe mitral regurgitation or reoperation for mitral regurgitation during a mean follow up postoperatively. We had for grade 1 a rate of 3.9 per 100 patient years for grade 2 1.86 per 100 patients years and for grade 3 the recurrence rate was 0.44 per 100 patients years, respectively. Our follow up for MVR group was only 80% completed unfortunately.

**Table 4:** Echocardiography control

**Discharge echocardiography for the total cohort**			
Presence of MR	MPL	MVR	Total
No MR	165	136	298
Grade 1 MR	37	4	41
Grade 2 MR	12	1	13
Grade 3 MR	0	0	0
**Total late echocardiography at follow up**			
Presence of MR	MPL (100 event/pat.yrs)	MVR (100 event/pat.yrs)	Total (100 event/pat.yrs)
No MR	144	108	252
Grade 1 MR	44 (3.91)	2 (0.27)	46(2.47)
Grade 2 MR	21 (1.86)	4 (0.54)	25(1.34)
Grade 3 MR	5 (0.44)	0 (0)	5 (0.27)
Follow-up pat.years	1125	734	1859

## Discussion

The main findings of this study are 1) that long term survival in patients operated with MPL was significant better than MVR, 2) no significant difference between the two groups was seen in thrombo-embolic episodes or bleeding and 3) the need for reoperation was not different between the MPL and MVR groups for patients with atrial fibrillation. However, patients operated on sinus rhythm with MPL not only lived longer compared to patients receiving mitral prosthesis, but also had a lower risk for reoperation.

Comparative studies of MPL/MVR are mostly observational retrospective cohort including our study. Unfortunately there is no until nowadays a randomised clinical trial comparing the 2 surgical procedures.

There is a variability as far inclusion of patients in those study as the factors determining the timing of surgery. We know that patients with left ventricular EF > 60% and left ventricular systolic diameter < 45 mm are under medical conservative treatment with ACE inhibitors and sometimes with beta blockers. In a symptomatic patient with EF < 60% and left ventricular end systolic diameter > 45 mm, MPL is indicated when MPL is expected to be durable. In the symptomatic patient with EF > 30% and left ventricular end systolic diameter < 55 mm the MR patients are candidates for MPL/MVR. (http://www.escardio.org/guidelines). (The new 2012 ESC/EACTS Guidelines on the management of valvular heart disease).

On the other hand there is also variability as far expertise of MPL in the major centres. Despite a consensus in guidelines [19] encouraging MPL, it is interesting to know that a significant number of patients with degenerative mitral valve disease still undergo planned mitral valve replacement all over the world. Many patients are operated with MVR despite repairable mitral valve pathology because surgeons who do not have the expertise to complete a successful surgical repair given the aetiology of degenerative disease could operate them.

### Endpoint survival

Our results showing a better survival in patients operated with MPL compared to MVR are in accordance with previous studies of cohorts with mixed aetiologies.

The major end point of those cohorts was survival (early and long term mortality) and survival free of re-operation and thromboembolism. The distributions of aetiology were: four degenerative myxomatous aetiology cohorts [20-23], four ischemic aetiology cohorts [24-27], while ten cohorts had mixed aetiologies [28-37]. Finally two important studies were with rheumatic aetiology [38,39]. In a meta analysis [1], the overall results of 21 studies as far survival pinpointed, a significant Hazard ratio =0.63 95% CL (0.36-0.71) indicating a 37% reduced risk of mortality for repair as compared to replacement whatsoever aetiology. However, by stratifying on aetiology, the beneficial effect on survival for repair compared to replacement was evident for degenerative/myxomatous, mixed and, rheumatic aetiologies. In patients with ischemic aetiologies, the results are not conclusive. Because of power limitation a subgroup analysis with stratification on aetiology in our study could not be performed.

Unfortunately when interpreting studies on long term survival it should be understood that available data refer to the outcomes of MPL and cardiac surgery as practiced 10 to 20 years previously. Cardiac surgery has since improved in several ways: for example, the widespread adoption of blood cardioplegia has likely reduced the ventricular damage during surgery, which in turn will impact long-term survival.

Therefore no way of knowing the long-term survival outcomes of mitral valve surgery as currently practised. Based on what is published, it appears that if surgery is undertaken before onset of symptoms and where left ventricular function is preserved, the life expectancy should be similar to that of the general population [40-42]. When significant symptoms of heart failure have developed NYHA-III or NYHA IV before mitral valve surgery is undertaken, the long-term survival is reduced, regardless of the left ventricular function [42].

### Endpoint survival free from re-operation, atrial fibrillation an effect modifier

In the literature we have found only 6 cohort studies permitting to estimate re operation hazard ratio for MPL compared to MVR [7,22,31,35,37,39]. The total summary hazard ratio of these studies HR =0.88 95% CL (0.48-1.62) suggests no difference in survival free from re operation between the procedures repair/replacement, and is in accordance with our study. The major reasons for re operations were technical failure and valve related causes such as infection, progression of disease and thrombosis. Unfortunately none of those studies has investigated and pinpointed the presence of AF as an effect modifier for the association between procedure and re-operation. The prevalence of AF at baseline was around 31% and it is well known that in the early phase of degenerative mitral disease treated conservatively the most frequent echocardiographic findings is the enlargement of the left atrium in patients on sinus rhythm. However paroxysmal or permanent AF ultimately affects 50% of patients within 10 years and after onset of AF an increased cardiac mortality and morbidity is observed. On the other hand patients who are surgically treated with preoperative AF had a worse postoperative outcome than those in Sinus Rhythm [41]. Grigioni F et al. [42] found that risk of AF increases with advancing age and larger LA dimension, in our study patients with AF at baseline were older had larger LA diameter and lower ejection fraction than patients in sinus rhythm. In concordance with Grigioni F et al. our results suggest that the clinical management of mitral disease should take into account the high incidence and excess risk of AF and more attention needs to be done in controlling its predictors. On the other hand both outcomes total mortality and re operation are not sensitive markers for evaluation of the efficacy of repair as compared to replacement. In fact failure of repair is mostly defined by recurrence of moderate or severe mitral regurgitation or re operation for mitral regurgitation. Failure rates of repair are determined principally by the original dysfunction and by the repair technique.

The longest data we have are about the conventional Carpentier technique, Baumberger et al. [43] Showed on patients with degenerative aetiology 97% of patients with posterior leaflet, 86% with anterior leaflet and 83.5% with bi leaflet prolapsed were free from re operation at 20 years.

They also found that only 74% were free from cardiac related events at 20 years. This difference between freedom from re operation and freedom from cardiac events, however pinpoint the limitations of outcome survival free from re operation as a good end point measure of the efficacy of MPL. Because of that we have enough evidence nowadays that survival and re operation rates are weak surrogates for adequacy of repair during the follow up time after mitral valve surgery.

### Importance of echocardiography follow up over time

Long term echocardiography follow up data are scarce in the medical literature, probably due to a high drop out rate of the patients as shown in long-term follow up study of MPL. David TE et al. [44] presented incomplete 50% drop out follow up date on echocardiographic follow up. Our study also suffered of 20% drop out mainly in the MVR group. The lack of systematic echocardiographic follow-up is the major limiting factor in determining the true durability of all MPL techniques [45]. Most cohort studies in the literatures [1] have focused on survival and re operation rate, which may not necessarily reflect the durability of repair. The most complete serial echocardiographic follow up data for MPL is from Flameng et al. [46]. They performed echocardiografic examinations in a serial follow up done at 6 months intervals like our protocol. After 7 years the, freedom from moderate or severe MR was 71%. New recurrent mitral regurgitation appeared at a rate of 3.7% per year. The recurrence rate per year was 8.3% for MR grade > I, and 3.4% for MR grade > II. These results are in accordance with ours showing a recurrence rate of 2.3% per year for MR grade ≥ II and pinpointing a MR recurrence rate of 2.30 per% per year for MR Grade > =II. Furthermore, our results are concordant with Flameng et al. and underline that durability of many MPLs is limited. The linear recurrence rate implies that recurrent mitral regurgitation probably reflects the progression of mitral valve disease, while technical failure is the major cause of early regurgitation [47].

## Conclusion

As a conclusion our results suggest a beneficial effect of MV-repair compared to MV-replacement on total mortality and re operation for patients on sinus rhythm. For patients with AF it is beneficial only for mortality. We advocate more attention in controlling risk factors of AF in the clinical management of mitral disease. Being in a non-randomised design our results need to be replicated to be conclusive by a randomised clinical trial with stratification on different aetiologies and on the presence of atrial fibrillation. The major end points of this trial will be the durability and adequacy of repair and the impact of repair on left ventricular systolic functions over time using a serial echocardiographic follow up.

## Consent

Written informed consent was obtained from the patient for publication of this report.

## Abbreviations

MPL: Mitral plasty; MVR: Mitral valve replacement; MR: Mitral regurgitation; MS: Mitral stenose; AF: Atrial fibrillation; AS: Aortic stenosis; TR: Tricuspidal regurgitation; AR: Aortic regurgitation; PS: Propensity score; SE: Standard error.

## Competing interests

The authors declare that they have no competing interests.

## Authors’ contributions

All authors: 1) have made substantial contributions to conception and design, or acquisition of data, or analysis and interpretation of data; 2) have been involved in drafting the manuscript or revising it critically for important intellectual content; and 3) have given final approval of the version to be published.
